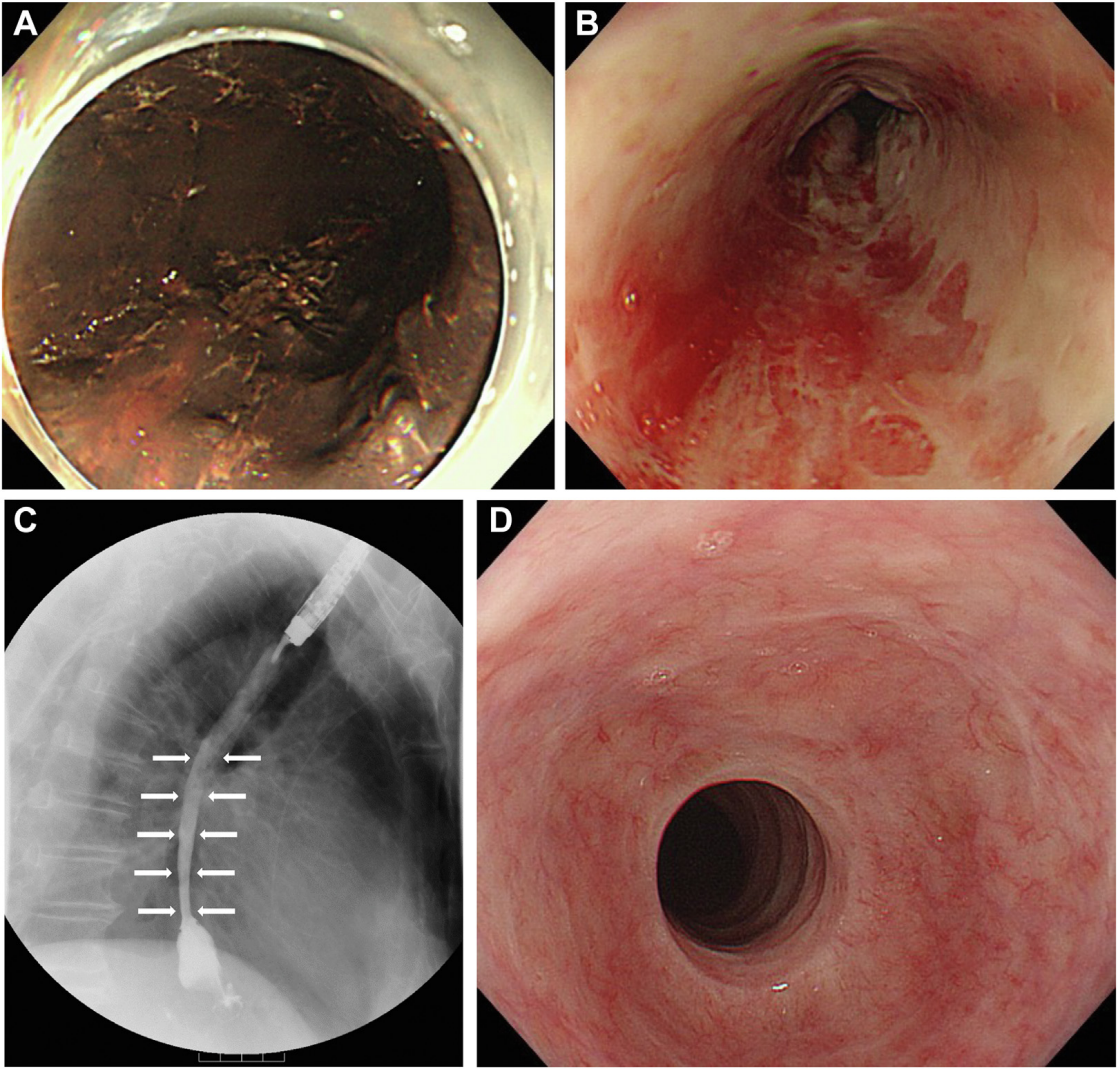# A Case of Acute Necrotizing Esophagitis With Effective Steroid Therapy for Stenosis Prevention

**DOI:** 10.1016/j.gastha.2022.05.012

**Published:** 2022-05-24

**Authors:** Kazuki Natsui, Masaki Maruyama, Shuji Terai

**Affiliations:** 1 Department of Gastroenterology, Kashiwazaki General Hospital and Medical Center, Kashiwazaki, Niigata, Japan; 2 Division of Gastroenterology and Hepatology, Graduate School of Medical and Dental Sciences, Niigata University, Chuo-Ku, Niigata, Japan

An 80-year-old man with a complaint of hematemesis was taken to our emergency department. Blood tests showed decreased hemoglobin, 12.6 g/dL (normal range, 13.7–16.8 g/dL), and increased D-dimer, 9.0 μg/mL (normal range, <1 μg/mL). Contrast-enhanced computed tomography revealed the edematous thickening of the entire esophageal wall and a peripheral pulmonary artery embolism. Esophagogastroduodenoscopy (EGD) showed a black appearance of the entire esophageal mucosa and easy bleeding only by passing of the scope (Figure A). Acute necrotizing esophagitis complicated by pulmonary embolism was diagnosed. The patient was treated with proton pomp inhibitor. On day 9, EGD and esophageal transit with gastrografin showed ameliorated bleeding tendency of the mucosa with moderate stenosis in the lower esophagus (Figure B and Figure C). Prednisolone (30 mg) was initiated orally in accordance with the preventative treatment for stenosis in corrosive esophagitis and circumferential endoscopic submucosal dissection for esophageal carcinoma. Prednisolone was tapered off and discontinued after 6 weeks. On day 16, EGD revealed improved stenosis. Six months after discharge, EGD showed a recovered normal esophageal mucosa with only a residual upper-esophagus ring-shaped scar (Figure D). Stenosis can also occur as an acute necrotizing esophagitis complication, and early steroid therapy is a possible treatment option.

**Figure:**